# *IGF1* gene polymorphisms associated with diabetic retinopathy risk in Chinese Han population

**DOI:** 10.18632/oncotarget.21366

**Published:** 2017-09-28

**Authors:** Jian Zhang, Xiao Chen, Like Zhang, Yi Peng

**Affiliations:** ^1^ Department of Endocrinology, Xingtai People's Hospital, Xingtai 054031, Hebei, China; ^2^ Department of Medical Optometry, Ophthalmology Hospital, Xingtai 054001, Hebei, China; ^3^ Department of Endocrinology, The First People's Hospital of Zhangjiakou, Zhangjiakou 075000, Hebei, China

**Keywords:** *IGF1*, diabetic retinopathy, t2dm, haplotype, serum concentration

## Abstract

**Objective:**

This study aimed to explore the association of insulin-like growth factor 1 gene (*IGF1*) polymorphisms with diabetic retinopathy (DR) in a Chinese Han population.

**Methods:**

Polymerase chain reaction-restriction fragment length polymorphism (PCR-RFLP) was used for genotyping. Genotype frequencies were compared by chi-square test. Odds ratio (OR) with 95% confidence interval (95%CI) was calculated to express the risk intensity of DR. Linkage disequilibrium between *IGF1* polymorphisms was analyzed by Haploview. Serum IGF1 concentration was measured by enzyme-linked immunosorbent assays (ELISA) and assessed by student's t test.

**Results:**

AG genotype of rs6218 and TT genotype of rs35767 were significantly associated with the elevated risk of DR (rs6218: OR=1.77, *P*=0.04; rs35767: OR=2.32, *P*=0.03) and type II diabetes mellitus (T2DM) (rs6218: OR=1.92, *P*=0.00. rs35767: OR=2.29, *P*=0.02). Only T allele of rs35767 significantly increased the risk of DR (OR=1.45, *P*=0.04), however, rs6218 (OR=1.92, *P*=0.00), rs35767 (OR=0.02, *P*=0.02) and rs5742612 (OR=2.21, *P*=0.04) showed obvious association with T2DM. Haplotypes were only associated with T2DM, but not DR. Minor allele homozygote of rs35767 was obviously correlated with serum IGF1 level.

**Conclusion:**

*IGF1* rs6218 and rs35767 polymorphisms contribute to the risk of DR. *IGF1* rs35767 polymorphism may participate in the regulation of serum IGF1 concentration in DR.

## INTRODUCTION

Diabetic retinopathy (DR) is a serious microvascular complication of diabetes mellitus (DM). The disease is characterized by progressive retinal degeneration, representing a leading cause of blindness in adults [1–3]. According to epidemiological investigations, the prevalence of DM was 285 million in the world in 2010, over one third of cases had developed DR [4]. Almost all type I DM (T1DM) cases and over 60% type II DM (T2DM) patients finally develop DR [5]. DR is also found to be associated with the elevated risk of some life-threatening diseases, such as stroke, heart failure and coronary artery disease (CAD) [6]. So, investigating the pathogenesis of DR and symptomatic treatments are necessary and urgent nowadays. Poor control of blood glucose, blood pressure, diabetes duration are the important risk factors of DR development and progression [7, 8]. However, some DM patients with optimum glycemic control still develop DR [9], suggesting individually genetic factors are crucial elements of DR occurrence.

Insulin-like growth factor 1 (IGF1) is an important growth factor associated with multiple biologucal systems, such as cell proliferation, differentiation, survival and maturation [10]. It is usually synthesized and secreted by liver cells and exerts function via binding to the specific receptor IGF1R [11]. In structure, IGF1 is highly homologous with pro-insulin, also including receptor. It is identified to contribute to the main pathways in the progression of DM complications by endocrine [12]. What's more, IGF1 is reported to be involved in the development of epiretinal membranes (ERMs) which are a layer of pathologic tissues and are very important pathological conditions for DR [13–15].

In recent years, single nucleotide polymorphism (SNP) has become an important mean of revealing the susceptibility of individuals to some diseases. IGF1 protein encoded gene, *IGF1* is located on chromosome 12q22-23 [16] and consisted of 5 exons and 4 introns, including a number of SNPs [17, 18]. However, few studies referred to the association between *IGF1* SNPs and DR occurrence. Therefore, in this study, our objective was to explore the role of *IGF1* polymorphisms in DR development in Chinese Han population and a total of four common SNPs in *IGF1* were selected, including rs972936 and rs6218, rs35767 and rs5742612.

## RESULTS

### Basic characteristics

Demographic characteristics and clinical parameters of the study groups were summarized in Table 1. Age and gender distributions had no significant difference between DR, diabetic non-retinopathy (DNR) and healthy control groups (*P*>0.05). Disease duration of DM was longer in DR patients than in DNR patients, despite the difference was not significant (*P*=0.343). Body mass index (BMI), systolic blood pressure (SBP), diastolic blood pressure (DBP) had marginally significant difference in DR vs. Control and DR vs. DNR comparison respectively (*P<*0.05). Fasting plasma glucose (FPG) had significantly higher level in DR patients than in controls (*P*=0.008), but no significant difference existed between DR and DNR patients. Besides, we failed to find any significant difference of other demographic characteristics and biochemical measures, such as smoking, drinking, triglyceride (TG), total cholesterol (TC), high density lipoprotein (HDL), low density lipoprotein (LDL) (*P*>0.05 for all).

**Table 1:** Baseline characteristics of participants

Characteristic	DR n=115(%)	DNR n=129(%)	Control n=142(%)	*P1*	*P2*
Age	59.72±11.38	59.05±10.88	57.9±10.61	0.235	0.264
Gender (male)	49(42.61)	54(41.86)	57(40.14)	0.689	0.906
Duration of DM (years)	14.05±7.72	13.98±8.67	-	-	0.343
Smoking (yes)	43(37.39)	43(33.33)	44(30.99)	0.281	0.508
Drinking (yes)	47(40.87)	49(37.98)	48(33.80)	0.243	0.645
BMI (Kg/m^2^)	22.3±6.8	23.5±6.5	24.5±6.3	0.034	0.042
SBP (mmHg)	140.6±20.25	135.8±19.77	127.6±19.42	0.035	0.047
DBP (mmHg)	81.72±12.39	81.96±11.96	79.81±11.90	0.057	0.107
FPG ((mmol/L)	10.19±4.39	8.43±3.57	7.85±4.23	0.008	0.098
TG (mmol/L)	1.59±1.12	1.65±1.37	1.61±1.23	0.302	0.221
TC (mmol/L)	4.16±1.79	3.87±1.86	4.24±1.91	0.351	0.168
HDL (mmol/L)	1.14±0.64	0.97±0.61	1.16±0.52	0.213	0.113
LDL (mmol/L)	2.22±1.31	2.06±1.24	2.19±1.27	0.863	0.761
IGF1 (mg/dL)	207.14±11.38	173.43±12.04	139.58±11.97	0.004	0.007

Notes: P1: DR vs. Control; P2: DR vs. DNR; BMI, body mass index; SBP, systolic blood pressure; DBP, diastolic blood pressure; FPG, fasting plasma glucose; TG, triglyceride; TC, total cholesterol; HDL, high density lipoprotein; LDL, low density lipoprotein; significant level was adjusted by Bonferroni method.

### Genotype distribution of *IGF1* polymorphisms in DR, DNR and healthy control groups

Genotype frequencies of *IGF1* polymorphisms in DR, DNR and healthy control groups were displayed in Table 2. Genotype distributions of *IGF1* polymorphisms were accorded with the HWE test in the control group. It demonstrated that the study subjects could present the same Mendelian population.

**Table 2:** The genotype distributions of *IGF1* polymorphisms in subjects

Genotype		DR		DNR		*P1*	OR (95%CI)	Control		*P*_HWE_	*P2*	OR (95%CI)
		N=115	%	N=129	%			N=142	%			
rs972936	CC	24	20.87	35	27.13	-	Ref.	37	26.06	0.07	-	Ref.
	CT	65	56.52	73	56.59	0.41	1.30 (0.70-2.41)	81	57.04		0.79	1.07 (0.65-1.75)
	TT	26	22.61	21	16.28	0.13	1.81 (0.83-3.92)	24	16.90		0.53	1.23 (0.65-2.33)
rs6218	AA	34	29.57	55	42.63	-	Ref.	75	52.82	0.62	-	Ref.
	AG	69	60.00	63	48.84	0.04	1.77 (1.03-3.06)	58	40.84		0.00	1.92 (1.24-2.97)
	GG	12	10.43	11	8.53	0.23	1.77 (0.70-4.44)	9	6.34		0.07	2.15 (0.94-4.94)
rs35767	CC	31	26.96	46	35.66	-	Ref.	56	39.44	0.12	-	Ref.
	CT	59	51.30	67	51.94	0.36	1.31 (0.74-2.32)	73	51.41		0.32	1.26 (0.80-1.97)
	TT	25	21.74	16	12.40	0.03	2.32 (1.07-5.03)	13	9.15		0.02	2.29 (1.13-4.68)
rs5742612	TT	41	35.65	56	43.41	-	Ref.	Ref.	48.59	0.39	-	Ref.
	CT	56	48.70	60	46.51	0.38	1.28 (0.74-2.19)	63	44.37		0.22	1.31 (0.85-2.02)
	CC	18	15.65	13	10.08	0.13	1.89 (0.83-4.29)	10	7.04		0.04	2.21 (1.01-4.80)

Notes: P1: DR vs. DNR; P2: DR+DNR vs. Control.

In our study, genotypes of rs972936 SNP had no significant difference between DR and DNR, DR+DNR and controls (*P*>0.05). It suggested that rs972936 SNP was not associated with the risk of T2DM or DR occurrence. Heterozygous AG genotype of rs6218 was significantly associated with the elevated risk of DR development (OR=1.772, 95%CI=1.03-3.06), meanwhile, it was also a risk factor for occurrence of T2DM (*P*=0.00, OR=1.92, 95%CI=1.24-2.97). TT genotype of rs35767 carriers were significantly more in T2DM patients than that of the healthy controls (*P*=0.02, 16.80% vs. 9.15%), indicating it was a risk factor of T2DM (OR=2.29, 95%CI=1.13-4.68). Similarly, TT genotype frequency of rs35767 in DR group was obviously higher than that in DNR group (*P*=0.03, 21.74% vs. 12.40%). So it could significantly increase the risk of DR development, compared with CC genotype (OR=2.32, 95%CI=1.07-5.03). We also found that CC genotype frequency of rs5742612 between T2DM patients and the controls was significant different (*P*=0.04), but not DR or DNR (*P*=0.13). It indicated that CC genotype was associated with T2DM susceptibility (OR=2.21, 95%CI=1.01-4.80), but not DR.

### Allele distribution of *IGF1* polymorphisms in DR, DNR and healthy control groups

For allele of *IGF1* polymorphisms (Table 3), we found that only T allele of rs35767 had significantly higher frequency in DR than that in DNR groups (*P*=0.04, 47.39% vs. 38.37). When compared with C allele (52.61% vs. 61.63%), T allele might is a risk factor for DR occurrence (OR=1.45, 95%CI=1.01-2.08). But, alleles of rs972936, rs6218 and rs5742612 SNPs had no significant association with the DR susceptibility (*P*>0.05). Meanwhile, we also failed to detect the association between rs972936 allele with DR or T2DM development (*P*=0.57). *IGF1* rs6218 G allele (OR=1.57, 95%CI=1.14-2.12), rs35767 T allele (OR=1.39, 95%CI=1.03-1.88) and rs5742612 C allele (OR=1.39, 95%CI=1.02-1.91) may obviously enhance the risk of T2DM development.

**Table 3:** The allele distributions of *IGF1* polymorphisms in subjects

Genotype		DR		DNR		*P1*	OR (95%CI)	Control		*P2*	OR (95%CI)
		N=115	%	N=129	%			N=142	%		
rs972936	C	113	49.13	143	55.43	-	Ref.	155	54.58	-	Ref.
	T	117	50.87	115	44.57	0.16	1.29 (0.90-1.84)	129	45.42	0.57	1.09 (0.81-1.46)
rs6218	A	137	59.57	173	67.05	-	Ref.	208	73.24	-	Ref.
	G	93	40.43	85	32.95	0.09	1.38 (0.96-2.00)	76	26.76	0.01	1.57 (1.14-2.12)
rs35767	C	121	52.61	159	61.63	-	Ref.	185	65.14	-	Ref.
	T	109	47.39	99	38.37	0.04	1.45 (1.01-2.08)	99	34.86	0.03	1.39 (1.03-1.88)
rs5742612	T	138	60.00	172	66.67	-	Ref.	201	70.77	-	Ref.
	C	92	40.00	86	33.33	0.13	1.33 (0.92-1.93)	83	29.23	0.04	1.39 (1.02-1.91)

Notes: P1: DR vs. DNR; P2: DR+DNR vs. Control.

### Haplotype analysis between *IGF1* polymorphisms in DR occurrence

In the present study, the status of linkage disequilibrium in *IGF1* polymorphisms was analyzed. Strong linkage disequilibrium was detected between rs972936 and rs6218, rs35767 and rs5742612 (Table 4). Three haplotypes were found in rs972936-rs6218, namely, C-A, T-A and T-G haplotypes. However, we could not found any haplotype which was associated with the risk of DR occurrence (*P*>0.05). T-A (OR=0.62, 95%CI=0.45-0.95) and T-G (OR=1.42, 95%CI=1.02-1.98) haplotypes showed significant association with the susceptibility of T2DM occurrence, when compared with C-A haplotype. Strong linkage disequilibrium was also found between rs35767 and rs5742612. Only T-C haplotype was associated with the significantly increased risk of T2DM (OR=1.42, 95%CI=1.03-1.95), but any haplotypes of rs35767-rs5742612 did not associated with the susceptibility of DR development.

**Table 4:** The haplotype analysis between *IGF1* polymorphisms in DR

Haplotype	DR		DNR		*P1*	OR (95%CI)	Control		*P2*	OR (95%CI)
	2N=230	%	2N=258	%			2N=284	%		
rs972936-rs6218										
C-A	113	49.13	143	55.43	-	Ref.	155	54.58	-	Ref.
T-A	24	10.43	30	11.63	0.97	1.01 (0.56-1.83)	53	18.66	0.03	0.62 (0.40-0.95)
T-G	93	40.44	85	32.94	0.10	1.39 (0.94-2.03)	76	26.76	0.04	1.42 (1.02-1.98)
rs35767-rs5742612										
C-T	121	52.61	159	61.63	-	Ref.	185	65.14	-	Ref.
T-C	92	40.00	86	33.33	0.08	1.41 (0.96-2.05)	83	29.23	0.03	1.42 (1.03-1.95)
T-T	17	7.39	13	5.04	0.16	1.72 (0.80-3.67)	16	5.63	0.51	1.24 (0.66-2.34)

Notes: P1: DR vs. DNR; P2: DR+DNR vs. Control.

### Influence of *IGF1* polymorphisms on serum IGF1 concentration

**Figure 1:**
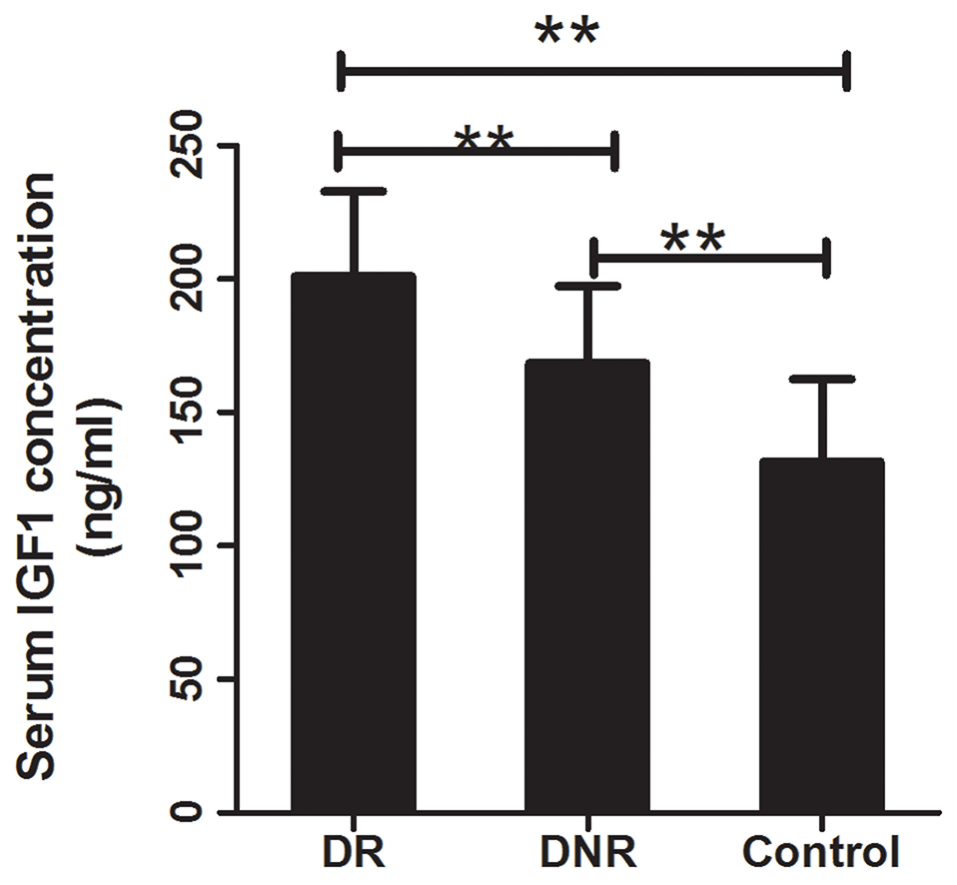
Serum concentration of IGF1 in DR, DNR and healthy control Compared with the healthy controls, serum concentration of IGF1 was significantly increased in DR and DNR groups (*P*<0.01 for both). Moreover, DR exhibited obviously increased serum IGF1 concentration compared to the DNR group (*P*<0.01). ^**^: indicated *P*<0.01.

**Figure 2:**
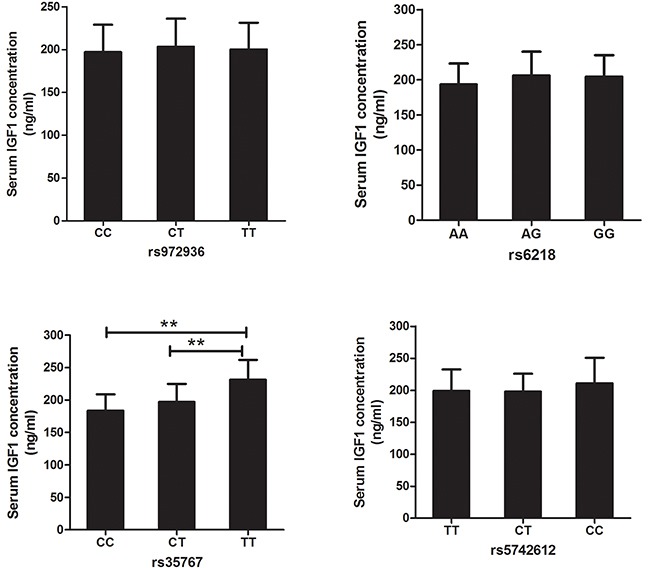
Association between *IGF1* polymorphisms and serum IGF1 concentration in DR Analysis result demonstrated that serum IGF1 concentrations didn't show significant difference between any two genotypes of rs972936, rs6218, or rs5742612 polymorphisms. Obvious higher serum IGF1 concentration was found in TT, compared with CT and CC genotypes carriers. Moreover, serum concentration of IGF1 was similar between CC and CT genotype carriers (*P*=0.0748). ^**^: suggested *P*<0.01.

For exploring the mechanism of *IGF1* polymorphisms in DR, we measured the influence of *IGF1* polymorphisms on serum IGF1 concentration. Student's t test demonstrated that compared with healthy controls, DR and DCR exhibited significantly elevated serum levels of IGF1 (*P*<0.01). Furthermore, serum concentration of IGF1 was obviously higher in DR than that in DCR (*P*<0.01) (Table 1, Figure 1). In DR patients, TT genotypes of rs35767 carriers had significantly higher serum IGF1 concentration than that of CC and CT genotype carriers respectively (*P*<0.01 for both, Figure 2). Serum IGF1 level was similar between CC and CT genotype carriers. In addition, serum IGF1 levels were similar between rs972936 CC, CT and TT genotypes. Similar results were observed in other two polymorphisms, that is, no significant difference of serum IGF1 concentration was found between any two genotypes of rs6218 or rs5742612 polymorphisms (Figure 2).

## DISCUSSION

Genetic factors play important roles in etiology of DR. SNP technology may be a promising approach to identify the susceptible population for DR, thus contributing to prevention and treatment of the disease. In the current study, we explored the genetic association of *IGF1* polymorphisms with DR development in Chinese Han population, as well as the related mechanisms. The results showed that AG genotype of rs6218 was obviously associated with the increased risk of DR and T2DM occurrence. TT genotype of rs35767 might also be a risk factor for DR and T2DM. CC genotype of rs5742612 was only associated with the obviously increased risk of T2DM, but not DR. In allele distribution, only rs35767 T allele was significantly correlated with the elevated DR susceptibility and T2DM risk. G allele of rs6128 and C allele of rs5742612 only showed significant associations with increased susceptibility for T2DM occurrence, but not DR. The alleles and genotypes of rs972936 was not distinctly correlated with DR or T2DM. The present study might point out a novel approach for identification of high risk population of DR, which could help improve the prevention and management of DR.

The role of *IGF1* polymorphisms in DR development had been reported in some populations. For example, in the study performed by Bazzaz et al., *IGF1*-383C/T and -1089C/T polymorphisms did not show significant association with DR susceptibility [12]. Uthra *et al*. investigated the role of cytosine-adenine (CA) [17,18] repeats in *IGF1* promoter region in DR development based on a Southern Indian cohort. The study showed that CA [18] repeats genotype was a risk factor of DR [19]. In this study, we found that rs35767 and rs6218 polymorphisms showed close association with risk of DR. In addition, strong linkage disequilibrium has been observed between rs972936 and rs6218, rs35767 and rs5742612. T-A and T-G haplotypes of rs972936-rs6128 were not related to DR susceptibility. However, the T-A haplotype might act as a protective factor and the T-G haplotype was a risk factor for T2DM. Similarly, T-C haplotype in rs35767-rs5742612 only contributed the risk of T2DM, but not DR susceptibility. Based on the data, we might reveal that the study polymorphisms might interact in the initiation and progression of DR.

Abnormal expression of IGF1 was observed in T2DM and DN cases, suggesting its functional roles in development and progression of the diseases [15, 20]. It was reported that IGF1 and glucose exposure could significantly promote migration and proliferation of retinal pigment epithelium *in vitro* [21]. In our study, we found that the expression patterns of IGF were significantly different among DR, DNR and the control groups, which was consistent with the previous investigations. In order to investigate the regulatory mechanisms of IGF1 polymorphisms in progression of DR, we measured the association of serum IGF1 concentration with the study polymorphisms in DR cases. Analysis results demonstrated that serum IGF1 concentration was significantly different between DR patients carrying TT, TC, CC genotypes of rs35767. Based on the data we speculated that rs35767 might influence DR susceptibility via altering the expression of *IGF1*. However, we couldn't find the obvious difference of serum IGF1 concentration in different genotypes of rs972936, rs6218 and rs5742612 polymorphisms respectively. Given the strong linkage disequilibrium between rs35767 and rs5742612, we speculated that rs972936, rs6218 and rs5742612 polymorphisms might be involved in development of DR via influencing the allele frequencies of rs35767 polymorphisms. Besides, the variants in IGF1 gene might able to regulate the activity of IGF1 protein, thereby taking part in the development and progression of DR. However, this assumption need to be verified in the future studies.

Differently, Broniarczyk *et al*. found that rs35767, rs5742612 polymorphisms in promoter region of *IGF1* didn't affect serum IGF1 level in children with growth disorders [22]. Kanbur and Sesti et al. reported that rs35767 was associated with changes of IGF1 level [23, 24]. In the study which carried by Li *et al*., rs6218 in 3′UTR of *IGF1* might regulate the transcriptional activity of *IGF1* mRNA level [25]. C allele of rs5742612 was also found to be associated with the increased circulating IGF1 level [26, 27]. These inconsistent results may result from the influence of disease microenvironment on genotype distribution of polymorphisms, different study populations or sample size.

There were several limitations in the current research. First, the sample size was relatively small in the current study. Second, the results obtained in our study might be limited by the single population. In the current study, all the study subjects belonged to Chinese Han population. Third, environment factors and other genetic factors did not considered in this study. Besides, present results did not adjusted by confounding factors. Additionally, we did not provide direct evidences for the regulatory mechanisms of *IGF1* polymorphisms in DNR initiation. Animal models or cell experiments might be applied to investigate the related regulatory mechanisms. Further investigations were still required to address the above issues.

In conclusion, *IGF1* rs6218 and rs35767 polymorphisms were significantly correlated to the occurrence risk of DR and T2DM, rs5742612 only influences the susceptibility to T2DM in this Chinese Han population. Rs35767 may participate in DR development through regulating serum IGF1 level, but not rs6218.

## MATERIALS AND METHODS

### Participants

In this study, a total of 244 type 2 diabetes mellitus (T2DM) patients (outpatients and inpatients) were recruited from the Department of Medical Optometry of Ophthalmology Hospital from 7, 2014 to 4, 2016, with the mean age of 59.6±11.3 years. They were all confirmed as T2DM according to the diagnosis criteria of WHO (World Health Organization) in 1999, including 103 males and 141 females. Among them, 115 patients had developed DR and the rest 129 were diabetic non-retinopathy (DNR) cases. DR patients were diagnosed by professional eye doctor through the careful examination in eyes. The disease duration of all T2DM patients were over 5 years. The participants would be excluded if they suffered from known other types diabetes, thyroid dysfunction, chronic kidney disease, anemia, mental illness. Furthermore, the pregnant or breastfeeding women were also not permitted in this investigation [28]. 142 healthy controls who visited the hospital for physical examination were also recruited in this study, including 57 males and 85 females with the average age of 57.9±10.61 years old. Healthy controls were matched with T2DM patients in age and gender. All participants were Chinese Han population without any blood relationship. This research was supported by the Ethics Committee of Ophthalmology Hospital. Before blood sample collection, written informed consents were signed by each participant.

### Sample collection and DNA extraction

After fasting for 8-10h, 2ml peripheral venous blood was collected from each participant in the early morning. The collected blood specimens were put in sterile and specified blood collection tube with EDTA, then plasma and leukocytes were isolated via centrifugation. The former was used to measure IGF1 concentration. Genomic DNA was extracted using TIANamp Genomic DNA Kit (TIANGEN BIOTECH (BEIJING) CO., LED), according to the manufacturer's instructions. Quality and concentration of DNA were detected by 1.0% agarose gel electrophoresis (AGE) and NanoDrop 2000c.

### Genotyping of *IGF1* polymorphisms

Polymerase chain reaction-restriction fragment length polymorphism (PCR-RFLP) was used for genotyping of *IGF1* polymophisms. PCR primers (Table 5) were designed by Primer Premier 5.0 software and synthesized in Sangon Biotech (Sangon, Shanghai). A volume of 25.0μl PCR system was used and PCR procedures were as follows: predenaturation at 95°C for 5min, 30-35 cycles with denaturation at 95°C for 30s, annealing at 56°C for 30s, extension at 72°C for 30s, and final extension at 72°C for 7min. PCR products were detected by 1.0% AGE.

**Table 5:** The primer sequences of *IGF1* polymorphisms

		Primer sequence	Position	Restriction enzyme
rs972936	For.	5′GTTTGAGTCTTTTCAAGCGTTCA3′	Intron2	*Dra* I
	Rev.	5′ATCTGGGTTAGTCATCTGTGGCA3′		
rs6218	For.	5′TCTGTGGAATAAGATACTGGACT3′	3′UTR	*Dra* I
	Rev.	5′ATCTAACTATGACAGAAAACACG3′		
rs35767	For.	5′GAGCCAGAGTAGGATTTCAAGCA3′	Promoter	*Hinf* I
	Rev.	5′CCAGAGCAGACATACCTCTTTCC3′		
rs5742612	For.	5′AAAGTATGAGACAGTGCCCTAAA3′	Promoter	*Ava* I
	Rev.	5′GGTAAAGTAGATTGGAAGACAGC3′		

Note: 3′UTR: 3′ untranslated region.

Then PCR products were digested by specific restriction enzyme (Table 5). Enzyme-digested products were separated by 2.0% AGE.

### Measurement of serum IGF1 concentration

Serum IGF1 concentrations in DR, DNR and the healthy control groups were measured by enzyme-linked immunosorbent assays (ELISA) using Human IGF-1 Elisa Kit (Shanghai Enzyme-linked Biotechnology Co., Ltd.). The operation procedures were according to the manufacturer's instruction. In the meanwhile, the differences of serum IGF1 concentrations were compared among different genotypes of *IGF1* polymorphisms.

### Statistical analysis

Genotype frequencies of *IGF1* each polymorphism were gained by direct counting. Genotype distribution of polymorphisms in the healthy control groups was tested whether was consistent with Hardy-Weinberg equilibrium (HWE). Significant differences of genotype, allele frequencies were compared among groups by chi-square test. Furthermore, the status of linkage disequilibrium between *IGF1* polymorphisms was examined by Haploview software. Odds ratio (OR) with corresponding 95% confidence interval (95%CI) was calculated to express the association between the risk of DR and T2DM and genetic variants in *IGF1*. Continuous variables were presented by mean±SD and analyzed by student's T test. The statistical analysis was performed in SPSS 18.0 software. *P*<0.05 was considered as statistically significant.
